# HER2/neu Expression in Low-Grade Serous Ovarian Carcinoma: A Pilot Two-Center Retrospective Study

**DOI:** 10.3390/jcm15031038

**Published:** 2026-01-28

**Authors:** Alina Badlaeva, Aleksandra Asaturova, Aleksandra Rogozhina, Larisa Ezhova, Natalia Arzhanukhina, Anna Tregubova, Dmitry Rogozhin, Gennady Sukhikh

**Affiliations:** 1 National Medical Research Center for Obstetrics, Gynecology and Perinatology Named After Academician V.I. Kulakov of the Ministry of Health of Russia, Moscow 117513, Russiadr.a.tregubova@gmail.com (A.T.);; 2 Department of Pathology and Clinical Pathology, Pirogov Russian National Research Medical University, Moscow 117997, Russia; 3 N.N. Blokhin National Medical Research Center of Oncology, Moscow 115522, Russia; natasha-toty1@mail.ru

**Keywords:** low-grade serous ovarian carcinoma, HER2/neu, ERBB2, target therapy

## Abstract

**Background/Objectives**: Low-grade serous ovarian carcinoma (LGSOC) is known as an uncommon subtype of cancer with poor response to standard chemotherapy, so novel targets are required. The current study aims to highlight the Human Epidermal Growth Factor Receptor-2 (HER2/neu) immunohistochemical (IHC) expression in LGSOCs. **Methods**: The study was conducted using 33 cases of LGSOCs from the calendar years 2017–2024. IHC staining was performed using antibody anti-HER2/neu (clone 4B5). HER2/neu scoring was performed based on the American Society of Clinical Oncology and the College of American Pathologists (ASCO/CAP) criteria for breast carcinoma. **Results**: The mean age of the 33 patients was 46.5 years. International Federation of Gynecology and Obstetrics (FIGO) stage data for patients revealed a predominance of advanced disease: 82.7% (24/29) were in advanced stages. Early stages comprised 17.3% (5/29) of cases. The study did not find HER2/neu overexpression in all cases. In 3.0% of samples (1/33), HER2/neu IHC staining was scored as 1+, and in 6.1% (2/33) of all LGSOCs, ultralow phenotype was observed. Of 23 cases in the HER2-negative group, 6 patients were alive with progressive disease, 1 patient died in 5 months, and 16 were alive with no evidence of disease. Of two patients with the HER2-ultralow phenotype, one was alive with no evidence of disease at 16 months follow-up. **Conclusions**: The results support the idea that HER2/neu overexpression is exceptionally rare in LGSOC; nevertheless, future trials are essential to fully characterize the spectrum of HER2/neu alterations in LGSOC and to determine definitively whether the rare cases with mutations or ultralow expression could represent a small subgroup that might benefit from specific targeted agents.

## 1. Introduction

Low-grade serous ovarian carcinoma (LGSOC) is a rare subtype of ovarian cancer with distinct histopathological and molecular characteristics, as well as a unique clinical course [1]. Compared to the more common high-grade serous ovarian carcinoma (HGSOC), this slow-growing tumor is diagnosed at a younger age and is associated with favorable outcomes, but it shows resistance to platinum-based chemotherapy [2].

Histologically, LGSOCs display minimal or moderate nuclear atypia and low proliferative and mitotic activity. However, this biological potential has a dual effect on the clinical course: indolent growth is linked to tumor detection at an early stage, which may contribute to increased overall survival [3]. On the other hand, low proliferative activity also underlies the resistance of this tumor histotype to platinum-based chemotherapy regimens. Recent reports indicate that the response rate to first-line therapy ranges from 4.0% to 23.1%, for platinum re-challenge from 2.1% to 4.9%, and for paclitaxel monochemotherapy from 9.1% to 15.5% [4,5].

Several studies have shown that LGSOC may rarely harbor Erb-B2 Receptor Tyrosine Kinase 2 (ERBB2) alterations, which encodes Human Epidermal Growth Factor Receptor-2, also known as HER2/neu [6,7,8,9]. The HER2/neu proto-oncogene encodes a receptor tyrosine kinase of the Epidermal Growth Factor Receptor (EGFR) family (HER1-4) which, when activated, is involved in the activation of downstream signaling pathways important in tumorigenesis, such as unregulated cell proliferation and cell survival, making it a therapeutic target [6].

Currently, research is being conducted not only on drugs for HER2-positive cancer, but also for HER2-negative variants. For example, the drug trastuzumab deruxtecan (T-DXd) is effective against a type of metastatic breast cancer once considered “HER2-negative”. Clinical trials have shown it is effective for tumors with very low HER2 protein levels, now categorized as “HER2 low” and “HER2 ultralow” [8,10]. This has created a new treatment option for a large group of patients, leading to the approval of T-DXd for HER2-low metastatic breast cancer.

Although the HER2 protein is sometimes overexpressed, the ERBB2 signaling network and other tumor microenvironmental pathways, such as perineural invasion and microenvironmental tumor cell interactions—clinically described in the mechanisms of ovarian cancer—may be influenced by growth factor receptors [11,12]. Therefore, LGSOC is likely to be treated more effectively if both epithelial-acting oncogenes and the stromal and neural microenvironment that support and sustain tumor spread are considered.

Assessment of HER2 status has evolved as more has been learned about response interpretation, particularly regarding the HER2-low and -ultralow categories. Modern clinical and pathological diagnostic algorithms will be further adjusted to incorporate orthogonal molecular diagnostics, such as in situ hybridization (ISH) or next-generation sequencing (NGS), to resolve indeterminate immunohistochemical (IHC) assessments and to identify or classify certain genetic lesions that result in absence of protein overexpression [13].

Thus, knowledge about HER2 status in LGSOC is limited, and the available data are insufficient, often controversial, or describe only serous carcinoma without specifying the histological subtype. The current study aims to highlight HER2/neu IHC expression in LGSOCs.

## 2. Materials and Methods

### 2.1. Cohort Characteristics

Thirty-three cases of LGSOC were retrospectively collected for this study, with consent, from eligible female patients aged 30 to 83 years who underwent surgical treatment at two tertiary centers during the calendar years 2017–2024. The inclusion criteria were as follows: histologically confirmed diagnosis of LGSOC, patients over 18 years of age at the initial diagnosis, availability of representative formalin-fixed, paraffin-embedded (FFPE) tumor tissue blocks. The exclusion criteria were as follows: patients who underwent neoadjuvant chemotherapy, and inadequate or inappropriate tumor samples. Demographic, intraoperative, and clinical follow-up data were obtained from hospital and clinic charts.

### 2.2. Histological Method

Tumor samples were fixed in 10% neutral-buffered formalin with a cold ischemic time <1 h and fixation time of 6 to 48 h. After paraffin embedding, the samples were cut at 4 μm. For light microscopy, specimens were stained with hematoxylin and eosin (H&E) and independently evaluated by two gynecological pathologists (A.B. and A.A.) to confirm the diagnosis of LGSOC.

### 2.3. IHC Staining

To exclude pre-analytical factors in the two-institution cohort, central IHC staining for HER2/neu status was performed on recently cut 4 μm thick whole sections from FFPE blocks using the VENTANA UltraView DAB IHC Detection Kit (Ventana Medical-Systems, Oro Valley, AZ, USA) on an automated immunostainer BenchMark XT (Ventana Medical-Systems, Oro Valley, AZ, USA) using a locked protocol with rabbit monoclonal antibody anti-HER2/neu (clone 4B5, Roche, Basel, Switzerland). On-slide external breast cancer controls in every IHC run covering a full range of expression were used; internal controls in ovarian tissue were not applicable. For antigen retrieval, a CC1 solution was used in mild mode, and the antibody was incubated for 16 min at 37 °C. Peroxidase activity was detected by incubation in diaminobenzidine. The sections were then counterstained with haematoxylin, dehydrated in graded ethanols, cleared in xylene, and mounted under a coverslip for light microscopic examination.

### 2.4. HER2/neu Scoring

HER2/neu scoring was performed based on the American Society of Clinical Oncology and the College of American Pathologists (ASCO/CAP) criteria for breast carcinoma: score 0 for absent membrane staining; score 1+ for incomplete membrane staining that is faint/barely perceptible and within >10% of tumor cells; score 2+ for weak-to-moderate complete membrane staining in >10% of tumor cells or complete membrane staining that is intense but within ≤10% of tumor cells; score 3+ for complete membrane staining that is intense and within >10% of tumor cells. In addition to this, membrane staining that was incomplete, faint/barely perceptible, and in less than or equal to 10% of tumor cells was termed “HER2 ultralow” (score 0+). HER2 scoring was performed by a trained pathologist (A.B.); dual scoring, such as by two pathologists (A.B., A.A.), was performed for borderline HER2 scores to rereview and to provide consensus diagnosis. 

### 2.5. Statistical Methods

Statistical analyses were performed using GraphPad Prism 9.3.1 (Dotmatics, Boston, MA, USA). Quantitative indicators were assessed for normal distribution using the Shapiro–Wilk test. For indicators, the data were grouped into variation series, and the arithmetic mean (M), standard deviation (SD), and 95% confidence interval (95% CI) were calculated. Nominal data were described using absolute values and percentages. Patient survival analysis was performed using the Kaplan–Meier method, which enables the analysis of censored data, i.e., it estimates survival while accounting for the fact that patients may be lost to follow-up during the study or have varying observation periods. A *p*-value < 0.05 was considered significant.

## 3. Results

### 3.1. Immunohistochemical Analysis of HER2/neu Expression

**Figure 1:**
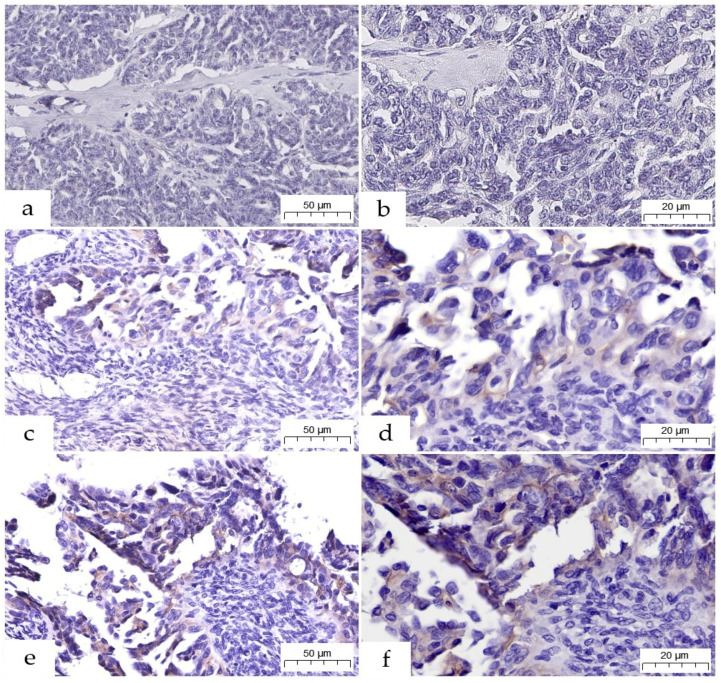
Variants of HER2-negative LGSOCs (previously unpublished, original photos). HER2 0 score with absent membrane staining of tumor cells (**a**,**b**); HER2 0+ score with incomplete faint membrane staining in less than 10% of tumor cells (**c**,**d**); HER2 1+ score with incomplete, barely perceptible membrane staining within >10% of tumor cells (**e**,**f**). IHC staining with monoclonal antibody anti-HER2/neu (original magnification 200× (**a**,**c**,**e**), 400× (**b**,**d**,**f**), resolution 0.12 µm/pixel).

Regarding the IHC analysis of the 33 LGSOC cases, no HER2/neu protein overexpression with scores of 2+ or 3+ was identified. All staining patterns were within the HER2-negative category. Almost all tumors, 90.9% (30/33), showed no membrane staining and were scored as HER2 0 (Figure 1a,b). A small number of cases showed low-level expression: 6.1% (2/33) of tumors had incomplete, faint membrane staining in ≤10% of tumor cells, described as the HER2-ultralow phenotype (score 0+, Figure 1c,d), and one case (3.0%) had faint or barely perceptible incomplete membrane staining in >10% of tumor cells, classified as 1+ HER2 (Figure 1e,f).

### 3.2. Clinicopathological Characteristics of the Cohort

The clinicopathological characteristics of the study group are presented in Table 1. The 33 patients included in the study had a mean age of 46.5 years, which is typical for this type of ovarian cancer [1]. International Federation of Gynecology and Obstetrics (FIGO) stage was known for 29 patients. Staging showed that most cases were advanced, with 62.0% (18/29) at stage III and 20.7% (6/29) at stage IV. Early-stage disease (stages I and II) was rare, accounting for 13.8% (4/29) and 3.5% (1/29), respectively. Staging for four patients was unavailable due to the absence of complete cytoreductive surgical procedures, which prevented adequate pathological examination of the omentum and peritoneum.

### 3.3. Correlation of HER2 Status with Clinical Follow-Up

Clinical follow-up data, with a mean duration of 46.7 months, were available for 24 of the 33 patients studied. In analyzing outcomes in the HER2-negative patients (score 0, n = 23), there was a variable course of disease. Of the 23 HER2-negative patients with available follow-up, 17.4% (4/23) were alive with documented evidence of progressive disease at their last follow-up (15–58 months). In 8.7% (2/23) of the patients, a local tumor recurrence was identified 181 and 342 months after the initial diagnosis (primary tumor in 2008, recurrence in 2023; primary tumor in 1995, recurrence in 2024, respectively). HER2/neu status in these cases was evaluated using the specimen from the recurrent lesion. One patient (4.3%, 1/23) died of the disease within 5 months. The majority of patients in this group (69.6%, 16/23) were alive with no evidence of disease, with a mean follow-up of 24.8 months (range: 5–71 months) for this subgroup. Regarding the rare low-expression phenotypes, one of the two patients with the HER2-ultralow phenotype was alive with no evidence of disease at 16 months post-diagnosis.

**Figure 2:**
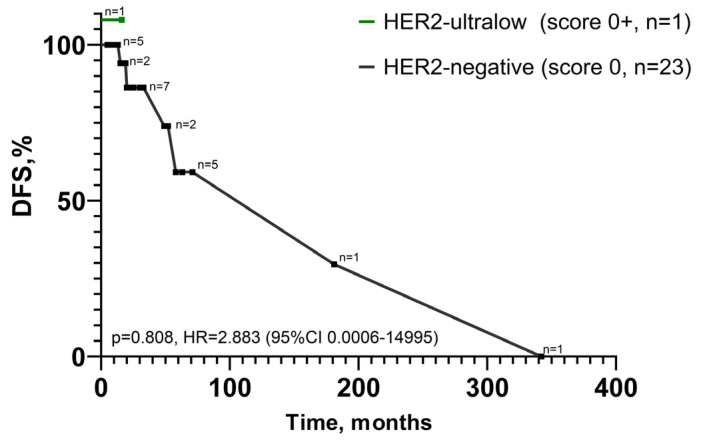
Univariable analysis of disease-free survival (DFS). DFS of cases with HER2-negative and -ultralow phenotypes. Labeled hazard ratio (HR) represents comparison of cases.

As can be seen from the Kaplan–Meier curves (Figure 2), analysis of disease-free survival did not identify any statistical differences between HER2-negative and -ultralow phenotypes (*p* = 0.808).

Follow-up data were unavailable for the second ultralow case and for the single patient with a HER2 1+ score, limiting further comparative analysis for these specific subgroups.

## 4. Discussion

LGSOC is known as an uncommon subtype of epithelial ovarian cancer with distinct clinical behavior and a specific molecular landscape, accounting for only 2–5% of all ovarian carcinomas [3]. Evidence suggests that most carcinomas subsequently develop after serous borderline tumors. A primary concern with LGSOC is its decreased sensitivity to standard platinum-based chemotherapy, and alternative treatments are still under development. Therefore, successful initial surgical reduction is especially important for LGSOC [6].

Due to the poor response to standard chemotherapy, novel therapeutic strategies are needed for patients with LGSOC. Recent data on signaling pathways have identified various oncogenic drivers of LGSOC [6,7]. These findings have led to promising clinical trials involving new targets, as highlighted in Table 2.

Mutually exclusive mutations in the upstream regulator genes of the mitogen-activated protein kinase (MAPK) pathway—B-type Rapidly Accelerated Fibrosarcoma *(BRAF)*, Kirsten Rat Sarcoma viral oncogene homolog *(KRAS)*, and Neuroblastoma Rat Sarcoma viral oncogene homolog *(NRAS)*—are the most prevalent genomic alterations in LGSOC [7,14]. In addition to the direct components of the MAPK signaling pathway, several related genes are overexpressed in low-grade tumors, including Neurofibromin 1 (*NF1)*, Neurofibromin 2 *(NF2)*, and Eukaryotic Translation Initiation Factor 1A X-linked *(EIF1AX)* genes. Recent studies indicate a synergistic relationship between NRAS mutations and those in *EIF1AX*, a gene involved in the initiation of protein translation. Mutations in the *EIF1AX* gene co-occur with those of *NRAS* and enhance proliferative and clonogenic capabilities when present together. This suggests a novel tumorigenic mechanism [15].

Research to date has investigated whether changes in the cyclin-dependent kinase inhibitor 2A *(CDKN2A)* gene, a cell cycle regulator, could also occur in LGSOC. These may involve gain or loss of the chromosomal region 9p containing the gene. The fact that *CDKN2A* loss is more common in malignant neoplasms than in borderline tumors indicates that loss of this cellular brake is an important step in progression from a less malignant to a more malignant state [16].

Recent studies have also reported mutations in non-MAPK pathway genes associated with LGSOC, including Ubiquitin Specific Peptidase 9, X-Linked *(USP9X)*, T-rich interaction domain 1A *(ARID1A)*, and phosphatidylinositol-4,5-bisphosphate 3-kinase catalytic subunit alpha *(PIK3CA)* [14,15,16]. The *USP9X* appears to be a specific driver of LGSOC, although its mode of action remains unknown and complex. Another gene, *ARID1A*, is an established to be a tumor suppressor in other cancers and is involved in chromatin remodeling to control gene expression [17].

Beyond the above-mentioned alterations, new mutations occurring in LGSOC have been identified by recent extensive genomic investigations, as previously mentioned. Promising case studies and clinical trials involving medications such as anastrozole, cyclin-dependent kinase 4 and 6 (CDK4/6) inhibitors, and EGFR inhibitors have resulted from these discoveries [14,15,16,17,18]. Nevertheless, because these mutations are relatively uncommon, they do not accurately represent LGSOC. Proliferation and activators of the MAPK pathway remain the main focus of current translational and clinical research, which has not yet produced the anticipated advances in treatment.

According to recent reports, anti-angiogenesis therapy, such as the anti-vascular endothelial growth factor (VEGF) monoclonal antibody bevacizumab, is a promising therapeutic option, demonstrating a median overall response rate of 47.5% when used alone or in combination with chemotherapy. This response rate is notably higher than the historically low efficacy of traditional chemotherapy (2.1–4.9%) or hormone therapy (9.0%) for this cancer [19,20]. The safety profile of bevacizumab in these studies was consistent with its known effects, including manageable risks of hypertension, gastrointestinal perforation, and wound healing complications. Despite these encouraging results, the available evidence is limited, consisting predominantly of small retrospective studies and one randomized trial subgroup with heterogeneous treatment regimens [19,20]. Consequently, the role of bevacizumab, particularly in the first-line setting, remains unclear and is not yet defined by robust evidence. Bevacizumab should be considered a viable treatment alternative for LGSOC, but there is an urgent need for prospective, well-designed clinical trials dedicated to this rare population to confirm efficacy and optimize its use.

So far, however, there has been little discussion about HER2 alterations in LGSOC. This proto-oncogene is an external regulator of the MAPK pathway, and mutations in these genes contribute to the activation of pathogenic pathways leading to ovarian cancer [21,22]. Pathological HER2 overexpression is defined relative to its low-level constitutive expression in normal cells, which have two gene copies and express approximately 40,000 HER2 molecules per cell. In HER2-positive breast cancer, defined by IHC 3+ or gene amplification on ISH, this gene is amplified to 25–50 copies. This genetic alteration leads to a corresponding 40–100-fold increase in HER2 protein, resulting in a tumor cell surface density of about 2 million receptors [23]. Therapeutically, this molecular signature provides a definitive target for pharmacological intervention against the HER2 oncogenic pathway, a strategy that has significantly improved patient prognoses.

Over the past four decades, the development of HER2-targeted therapies has achieved tremendous progress resulting in a wide range of agent classes, including monoclonal antibodies, antibody–drug conjugates (ADCs), and tyrosine kinase inhibitors (TKIs), which target the extracellular and intracellular domains of HER2, respectively [24]. With these therapies, there has been a dramatic improvement in clinical endpoints, with over 90.0% three-year survival in early breast cancer, as shown in [25]. However, a major clinical problem persists in the development of acquired resistance. This resistance is thought to arise through several biological mechanisms, including alterations in HER2 family members, activation of bypass pathways, and HER2 heterogeneity. As HER2 signaling is crucial for tumor growth, developing novel next-generation approaches to overcome resistance is an important clinical objective [26].

With the recent reaffirmation of the HER2 testing guidelines by ASCO/CAP, a significant milestone has been achieved in the understanding of breast cancer classification with the main focus derived from the practice-changing results of the DESTINY-Breast 04 trial [27]. This landmark study demonstrated a significant survival benefit with the HER2-targeting ADC T-DXd in patients with metastatic breast cancer exhibiting low levels of HER2 expression, a category now commonly termed “HER2 low.” Consequently, the U.S. Food and Drug Administration expanded its approval for T-DXd to include this new patient population [28,29].

This development represents a major paradigm shift, as it circumvents the long-standing dichotomous determination of HER2 status. Previously, tumors were strictly classified as HER2-positive or HER2-negative, which determined eligibility for anti-HER2 therapies. The new definition of HER2-negative transforms it into a therapeutically significant population. Consequently, the distinction between IHC 0 and IHC 1+ has gained considerable clinical relevance, as it now directly affects eligibility for a novel life-prolonging ADC [27,28,29]. However, this development introduces new diagnostic and therapeutic challenges. Data on tumors classified as IHC 0 are very limited, so it is unclear whether they benefit from newer ADCs. The current literature does not support IHC 0 versus 1+ as a new prognostic or predictive biomarker, but this analytical cutoff has become important [30,31,32]. Therefore, despite the conceptual prematurity of formally codifying new categories such as “HER2 low” and “HER2 ultralow” into clinical guidelines, the diagnostic reproducibility required to distinguish IHC 0 from IHC 1+ is now more critical than ever for optimizing patient selection and ensuring equitable access to this novel therapeutic strategy [28].

The current study did not find HER2/neu overexpression in all cases of LGSOC. However, IHC results showed heterogeneous staining patterns. In 3.0% (1/33) of cases, HER2/neu IHC staining was scored as 1+, and in 6.1% (2/33) of all LGSOCs, ultralow-HER2/neu phenotype was observed. In accordance with the present results, previous studies have demonstrated that LGSOCs were HER2/neu-negative. In a study which set out to determine novel candidate driver genes in LGSOC, Hunter et al. (2015) found that Sanger sequencing did not reveal mutations at exon 20 of the ERBB2 gene [33]. A recent study by Němejcová et al. (2025) involving HER2/neu IHC staining of 96 LGSOCs reported that HER2 expression (score 1+) was found in six cases, and HER2 ultralow expression was found in two cases [34]. Despite the large sample size, the main limitation of the above-mentioned study is the tissue microarray (TMA) approach, which could lead to underestimation or overestimation of the results, in contrast to the current study, where whole slides were analyzed to overcome the effects of intratumoral heterogeneity.

However, the findings of the current and previously described studies do not support the research by Bhardwaj et al. (2024), who identified HER2/neu IHC overexpression (score 2+ and 3+) in 5 of 24 cases of LGSOC, although 67.0% of samples were scored as 0 [21]. This research was limited by a lack of information on the HER2/neu antibody clone used and the absence of fluorescence ISH testing for samples with score 2+. Another earlier study reported “weak” HER2/neu IHC staining in five cases of well-differentiated serous carcinomas, which probably represent LGSOCs, so such results are difficult to compare [35]. The major limitations of this study are the small sample size, the lack of information on the HER2/neu scoring system, and the use of a polyclonal antibody, which is less specific. The data obtained in the current study also differ from the results of Stružinská et al. (2024) [36]. This study described the molecular landscape of 75 LGSOCs and found that 5.0% of cases harbored pathogenic or likely pathogenic *HER2/ERBB2* mutations.

Overall, these contradictory results may be due to a non-standardized IHC scoring system, the use of different antibody clones, and different technical approaches (TMA or whole section). This discrepancy in HER2/neu status could also be attributed to different types of analysis. In the current study, as well as in the studies by Němejcová et al. and Bhardwaj et al. [21,34], the IHC method detects protein overexpression, which is often caused by gene amplification rather than a single nucleotide mutation. Sanger sequencing, as used by Hunter et al. [33], identifies specific point mutations in the exons. It is entirely possible for a tumor to have a pathogenic mutation, as found by Stružinská et al. [36], that does not result in protein overexpression detectable by IHC.

Recent reviews on advanced diagnostic technologies highlight the growing importance of standardized, reproducible assays and integrated genomic profiling in oncology [37,38]. For LGSOC, future research must move beyond pilot IHC studies. To definitively characterize the HER2/ERBB2 landscape and its therapeutic relevance, well-designed prospective studies are needed. These studies should employ (1) harmonized IHC protocols and scoring criteria to ensure reproducibility, particularly for low-expression categories; (2) systematic orthogonal molecular testing (e.g., ISH for gene amplification, NGS for mutations) on IHC-negative and low-expressing cases to capture the full spectrum of alterations; and (3) larger, multi-institutional cohorts to achieve sufficient statistical power and generalizability. Such an approach will be crucial for identifying any small subset of LGSOC patients who might benefit from existing or future HER2-pathway-targeted agents.

Several limitations of this pilot study should be acknowledged. First, the obtained results are based on a small sample of LGSOCs partly due to its rarity [1,2,3]. Likewise, most of previous studies were also limited to a small number of cases [21,36,39]. In addition to this, a restricted retrospective cohort from two centers may introduce sampling bias and limit the statistical power and generalizability of our findings. Moreover, the data should be interpreted with caution because this investigation relies entirely on IHC without validation by genetic analysis such as ISH or NGS. This precludes definitive conclusions regarding the presence of ERBB2 gene amplification or specific activating mutations, which may occur independently of protein overexpression. Additionally, the current research was unable to analyze crosstalk between HER2/neu status and clinical follow-up.

The nonspecificity of HER2/neu protein positivity in our cohort, as documented by standard IHC criteria, has clinical implications. It suggests that HER2-directed therapies such as trastuzumab, which have been shown to be effective with high-level HER2 protein expression [13,40], will not be generally useful in the LGSOC population. The clinical implication of our findings is to assist in refining the therapeutic approach to this disease by utilizing alternative therapies for LGSOC (e.g., T-DXd), without including HER2 as a primary protein-based target for the vast majority of patients. Nevertheless, a cautious approach is warranted when assessing the clinical relevance of T-DXd for LGSOC. This is due to the lack of evidence supporting the extrapolation of response from HER2-low breast cancer without corroborative molecular and clinical trial data specific to LGSOC.

Nevertheless, the noted heterogeneity of limited expression requires careful consideration. The presence of a few cases with HER2-ultralow (0+) and 1+ phenotypes raises an important biological question. The clinical significance of HER2-low and -ultralow phenotypes, which have gained significance in breast carcinoma [13,29] with the advent of newer antibody-based therapeutics that appear effective in tumors with very limited HER2 expression, were not studied in this paper. The presence of these phenotypes suggests, at least in a small number of cases, a speculative area for further investigation in LGSOC. It remains to be seen whether such tumors exist as a biologically distinct group susceptible to next-generation targeted agents, which need to be evaluated in larger, prospectively designed trials.

The absence of HER2 protein overexpression in our cohort suggests that it is not a primary oncogenic driver in LGSOC. However, this does not preclude a role for the HER2 pathway or related receptor tyrosine kinases in modulating the tumor microenvironment. In other serous malignancies, pathways involving tumor–stroma and tumor–nerve crosstalk, such as those mediated by perineural invasion and tumor-associated Schwann cells, are key for invasion and may be regulated by receptors of the ERBB family [11,41]. Therefore, future molecular profiling of LGSOC should extend beyond epithelial cell mutations to include analyses of stromal and neural components. Investigating co-dysregulated pathways, such as those involving MAPK activation alongside microenvironmental remodeling signals, could reveal novel indirect therapeutic vulnerabilities.

Negative IHC results do not exclude a role for the HER2 pathway in LGSOC. Instead, they suggest a different therapeutic approach: targeting specific mutant isoforms rather than the receptor in bulk. Discrepancies in HER2 status across studies, such as those observed in non-small-cell lung cancer (where IHC 2+ concordance with ISH is only 9.1%) and in gastric cancer (where NGS and IHC show only moderate agreement), emphasize the need for standardized, reproducible assays in LGSOC. Harmonized IHC protocols, consensus scoring criteria, and routine use of ISH or NGS for validation are essential to clarify the true prevalence and therapeutic relevance of HER2 alterations in this rare tumor [42,43]. This implies that, in future, screening for molecular abnormalities in LGSOC should ideally include both IHC and NGS, as together they may capture the full spectrum of HER2/ERBB2 abnormalities, enabling identification of the small number of patients with actionable mutations for potential enrolment in clinical trials using TKIs or mutation-targeted agents.

## 5. Conclusions

The results of this research support the idea that HER2/neu protein overexpression is exceptionally rare in LGSOC, indicating it is not a dominant oncogenic driver in this subtype of ovarian cancer. However, these results should be interpreted with caution due to limitations such as the small cohort size, retrospective design, possible sampling bias, and reliance on IHC alone without validation through complementary genetic analysis. Future trials should assess the impact of HER2/neu expression in larger, uniformly processed, prospectively assembled cohorts. Incorporating parallel IHC and NGS is essential to fully characterize the spectrum of HER2/neu alterations in LGSOC and to determine definitively whether the rare cases with mutations or ultralow expression could represent a small subgroup that might benefit from specific targeted agents.

Comparative studies of breast, gastric, and non-small-cell lung cancers indicate that a comprehensive biomarker approach, incorporating IHC and orthogonal methods such as ISH or NGS, is necessary to optimally categorize patients. Negative IHC test results have treatment implications and do not exclude novel agents that target HER2-low and -ultralow cancers via antibody–drug conjugates. However, the use of these agents in LGSOC will require prior confirmation of HER2 protein expression at appropriate tissue concentrations, and preferably supporting evidence from specific clinical trial data.

More broadly, research on various cancer hallmarks is also needed to determine new, targetable mechanisms of carcinogenesis and development of LGSOCs. Beyond a limited analysis of the most frequent mutations (e.g., canonical MAPK signaling pathway), further studies on the role of other less common mutations, as well as the immune system, metabolism, epigenetics, non-coding ribonucleic acids (RNAs) (microRNA, long non-coding RNA, and circular RNA), and other malignant drivers in LGSOC would be worthwhile.

## Figures and Tables

**Table 1:** Clinicopathological features of LGSOC.

Characteristics	M ± SD (95% CI)	n, Total
Age (yr)	46.5 ± 12.4 (40–49)	33
Follow-up (mn)	46.7 ± 73.9 (15–49)	24 ^†^
FIGO stage	n (%)	n, total
I	4 (13.8)	29 ^††^
II	1 (3.5)	
III	18 (62.0)	
IV	6 (20.7)	
HER2 IHC	n (%)	n, total
0	30 (91.0)	33
0+	2 (6.0)	
1+	1 (3.0)	
2+/3+	0	

LGSOC, low-grade serous ovarian carcinoma; FIGO, International Federation of Gynecology and Obstetrics; IHC, immunohistochemistry; M, mean; SD, standard deviation; 95% CI, 95% confidence interval. ^†^ Follow-up data was not available for 9 patients; ^††^ FIGO stage was not available for 4 patients due to incomplete surgical staging.

**Table 2:** Potential therapeutic targets of LGSOC.

Target	Drug	Clinical Trial
Estrogen receptor	Aromatase inhibitor	NRG GY-019
	Aromatase inhibitor	ENGOT-ov54/MATAO
	Aromatase inhibitor	LEPRE
	Selective estrogen receptor degrader	FUCHSia Study
CDK4/6	CDK4/6 inhibitor	ENGOT-ov70/ALEPRO
BRAF	RAF inhibitor	ABM-1310
PD1	Checkpoint inhibitor	PERCEPTION
MEK1/2	Dual RAF/MEK inhibitor	GOG-3097/ENGOT-ov81/RAMP 301
	Dual RAF/MEK inhibitor	ENGOT-ov60/GOG-3052/RAMP-201
	Dual RAF/MEK inhibitor	MILO/ARRAY-162– 311/ENGOT-ov11
	MEK inhibitor	EMR 20 006–012

CDK4/6, cyclin-dependent kinase 4 and 6; PD1, programmed cell death 1; MEK1/2, mitogen-activated protein kinase 1 and 2; RAF, Rapidly Accelerated Fibrosarcoma.

## Data Availability

The original contributions presented in this study are included in the article. Further inquiries can be directed to the corresponding author.

## Abbreviations

The following abbreviations are used in this manuscript:

LGSOC	Low-grade serous ovarian carcinoma
HGSOC	High-grade serous ovarian carcinoma
ERBB2	Erb-B2 Receptor Tyrosine Kinase 2
HER2	Human Epidermal Growth Factor Receptor-2
EGFR	Epidermal Growth Factor Receptor
T-DXd	Trastuzumab deruxtecan
ISH	In situ hybridization
NGS	Next-generation sequencing
IHC	Immunohistochemical
FFPE	Formalin-fixed, paraffin-embedded
H&E	Hematoxylin and eosin
ASCO/CAP	American Society of Clinical Oncology and the College of American Pathologists
M	Arithmetic mean
SD	Standard deviation
95% CI	95% confidence interval
FIGO	International Federation of Gynecology and Obstetrics
HR	Hazard ratio
DFS	Disease-free survival
CDK4/6	Cyclin-dependent kinase 4 and 6
PD1	Programmed cell death 1
MEK1/2	Mitogen-activated protein kinase 1 and 2
RAF	Rapidly Accelerated Fibrosarcoma
MAPK	Mitogen-activated protein kinase
BRAF	B-type Rapidly Accelerated Fibrosarcoma
KRAS	Kirsten Rat Sarcoma viral oncogene homolog
NRAS	Neuroblastoma Rat Sarcoma viral oncogene homolog
NF1	Neurofibromin 1
NF2	Neurofibromin 2
EIF1AX	Eukaryotic Translation Initiation Factor 1A X-linked
CDKN2A	Cyclin-dependent kinase inhibitor 2A
USP9X	Ubiquitin Specific Peptidase 9, X-Linked
ARID1A	T-rich interaction domain 1A
PIK3CA	Phosphatidylinositol-4,5-bisphosphate 3-kinase catalytic subunit alpha
VEGF	Vascular endothelial growth factor
ADCs	Antibody–drug conjugates
TKIs	Tyrosine kinase inhibitors
TMA	Tissue microarray
RNAs	Ribonucleic acids
